# Supplementary material for: Protective Effect of Bifidobacterium animalis subs. lactis MG741 as Probiotics against UVB-Exposed Fibroblasts and Hairless Mice
Source: Microorganisms. 2022 Nov 26;10(12):2343. doi: 10.3390/microorganisms10122343 (PMC9782240; doi:10.3390/microorganisms10122343)
Supplement: Supplementary file 1 [file microorganisms-10-02343-s001.zip › microorganisms-2058236-supplementary.pdf]

Table S1. Primer sequence used in this study.

Origin	Gene[1]	Sequence (3′→5′)	
		Forward	Reverse
Human	*MMP-1*	GCATATCGATGCTGCTCTTTC	GATAACCTGGATCCATAGATCGTT
	MMP-3	CAAAACATATTTCTTTGTAGAGGACAA	TTCAGCTATTTGCTTGGGAAA
	GAPDH	ACCCACTCCTCCACCTTTG	ACCCACTCCTCCACCTTTG
Mouse	*IL-6*	GAGACTTCCATCCAGTTGCCT	TGGGAGTGGTATCCTCTGTGA
	TNF-	TGTCTACTCCTCAGAGCCCC	TGAGTCCTTGATGGTGGTGC
	GAPDH	TCTCCCTCACAATTTCCATCC	GGGTGCAGCGAACTTTATTG

[1]*MMP*, matrix metalloproteinase; *IL*, interleukin; *TNF*, tumor necrosis factor; *GAPDH*, glyceraldehyde 3-phosphate dehydrogenase.

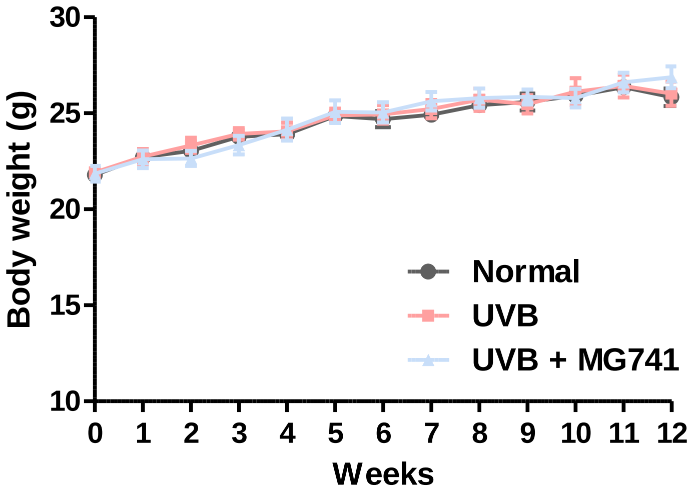

Figure S1. Body weight change against administration of *Bi. lactis* MG741 in UVB-exposed HR-1 mice. Data indicated mean ± SEM (n = 6).

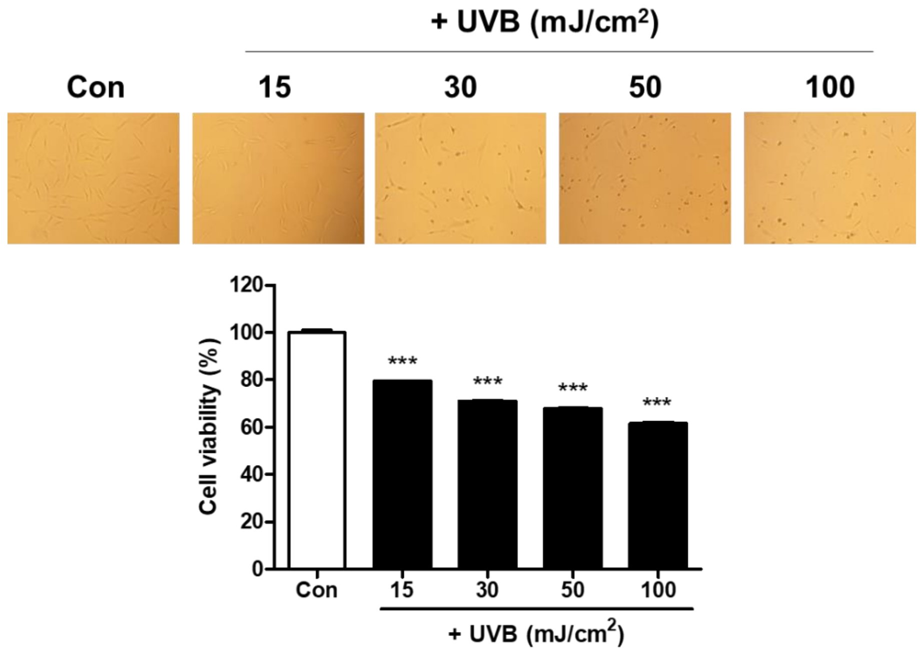

Figure S2. Cell morphology and viability against UVB treatment on Hs68 cells. UVB (15, 30, 50 and 100 mJ/cm^2) exposed Hs68 cells, for 24 h. The data indicated mean ± SEM in triplicate. Statistical differences are showed with ***$p < 0.001$ compared to control (non-expose UVB).
